# miR221 regulates cell migration by targeting *annexin a1* expression in human mesothelial MeT-5A cells neoplastic-like transformed by multi-walled carbon nanotube

**DOI:** 10.1186/s41021-021-00209-y

**Published:** 2021-08-02

**Authors:** Li Ju, Lijin Zhu, Hao Wu, Min Yu, Xianhong Yin, Zhenyu Jia, Lingfang Feng, Shibo Ying, Hailing Xia, Shuzhi Zhang, Jianlin Lou, Jun Yang

**Affiliations:** 1 Hangzhou Medical College, Hangzhou, 310013 China; 2 Jiading District Center for Disease Control and Prevention, Shanghai, 201800 China; 3 Hangzhou Normal University, School of Public Health, Hangzhou, 310036 China

**Keywords:** Multi-walled carbon nanotubes (MWCNT), Long-term exposure, Neoplastic-like transformation, *Annexin a1*

## Abstract

**Background:**

Multi-walled carbon nanotube (MWCNT) is one of the most widely used manufactured nanomaterials, however, its potential harmful effect on human health is of great concern. Previously we have shown the acute and chronic exposure to MWCNT induced different responses in human mesothelial MeT-5A cells. In the current study, MeT-5A cells were continuously subjected to MWCNT exposure at 10 μg/cm^2^ for 48 h per passage, up to a whole year, to further clarify the carcinogesis and its potential mechanisms of MWCNT.

**Results:**

After one-year MWCNT treatment, MeT-5A cells exhibited neoplastic-like properties, including morphological changes, anchorage-independent growth, increased cell proliferation and cell migration. Further examination revealed the expression of microRNA 221 (miR221) was gradually decreased, while the *annexin a1* expression was increased at both the mRNA and protein level during the exposure. Bioinformatic analysis indicated that *annexin a1* is a target for miR221 regulation, and it was confirmed by transfecting cells with miR221 mimics, which resulted in the downregulation of *annexin a1*. Detailed analyses demonstrated miR221 was involved in the regulation of cell migration, e.g., downregulation of miR221 or overexpression of ANNEXIN A1, contributed to the increased cell migration. In contrast, overexpression of miR221 or downregulation of ANNEXIN A1 slowed cell migration.

**Conclusions:**

Taken together, these results point to a neoplastic-transforming property of MWCNT, and the miR221-*annexin a1* axis is involved in the regulation of cell migration in the transformed cells.

**Supplementary Information:**

The online version contains supplementary material available at 10.1186/s41021-021-00209-y.

## Introduction

Multi-walled carbon nanotube (MWCNT) is one of the most widely used manufactured nanomaterials in many consumer and industrial products. MWCNT commonly found in the markets differs substantially in physico-chemical properties, including length, diameter, surface area, and surface chemistry, but all of them resemble asbestos, a Group 1 carcinogen classified by the International Agency for Research on Cancer (IARC), with the same high surface-volume ratio, especially the high aspect ratio [1]. Long-term, low dose MWCNT exposure, especially in occupational settings, is believed to have adverse effects to human health. Actually, accumulating evidence have shown clearly that exposure to MWCNT inflicts asbestos-like pathologic changes in experimental animals and strongly suggested that exposure to MWCNT may result in asbestos-like effects, such as lung carcinoma or pleural mesothelioma, in humans [2, 3]. As reported, intraperitoneal or intrascrotal injection of MWCNT caused mesothelioma at a high rate in rats, in which the surface-volume ratio and curvature seems to be an important parameter influencing the carcinogenicity of MWCNT [2, 4]. Intratracheal instillation of MWCNT-N induced both lung carcinoma and pleural mesothelioma in rats [5]. The induction of carcinomas and combined carcinomas and adenomas showed a dose-dependent manner in male rats exposed to MWCNT-7 after a 104-week inhalation [6]. In another whole-body inhalation studies, MWCNT-7 promoted methylcholanthrene-initiated lung carcinogenesis in mice. Inhalation of MWCNT-7 led to lung carcinoma in rats, and their lung burdens of MWCNT-7 increased with increasing concentration and exposure duration [5]. Based on the experimental animal data, MWCNT-7 has been classified as a possible carcinogen (Group 2B) for human by IARC.

However, the underlying mechanisms for MWCNT-induced carcinogenicity are not fully understood, although many hypotheses have been proposed and tested. For example, change of cellular oxidative status is recognized as a major mechanism [7]. In addition, MWCNT-induced aneuploidy (via centromere abnormalities), epithelial-mesenchymal transition (EMT), and neoplastic transformation, are all considered to be possible pathways contributing to carcinogenesis [8]. MWCNT-induced carcinogenesis may also involve ongoing low levels of DNA damage in an environment of persisting fibers, chronic inflammation and tissue irritation, and parallel increases or decreases in the expression of genes involved in several pro-carcinogenic pathways [9].

Nonetheless, carcinogenicity study is mostly conducted in animal models, which is not ideal for mechanistic study. As a consequence, various cell models have been utilized for the mechanistic study, including studies for MWCNT. Unfortunately, experiments conducted on cells usually last only several days or even hours, thus cannot reflect the long-term effect of MWCNT exposure. On the other hand, short-term or long-term exposure may have different effects on cells, thus could generate contradicting results. Indeed, as we had shown in a previous study, the response to MWCNT exposure for 72 h was different from the response for 30–90 days in human mesothelial MeT-5A cells [10]. Specifically, it was found that while exposure to MWCNT for 72 h decreased cell migration, longer exposure (30–90 days) partially reversed the effect, though still less than that of control cells. Furthermore, ANNEXIN A1 protein was shown to be involved in this process. ANNEXIN A1 has been shown to have various functions during tumor initiation and development in a tumor-specific manner. For instance, ANNEXIN A1 expression was prominently upregulated in papillary thyroid carcinoma (PTC) tumor tissues; furthermore, ANNEXIN A1 regulated EMT and activated the IL-6/JAK2/STAT3 pathway to contribute to PTC malignant behaviors, including PTC cell proliferation, migration and invasion [11]. In addition, increased ANNEXIN A1 expression is involved in prostate cancer cell migration which is regulated by androgen receptor signaling [12]. ANNEXIN A1 may also be involved in the pathogenesis of bone metastasis in small cell lung cancer (SCLC) [13]. However, how ANNEXIN A1 was regulated in our study is not clear. Nonetheless, in a microRNA (miRNAs) array study in these MWCNT-exposed cells, we identified a group of miRNAs with altered expression, including microRNA 221 (miR221). MiRNAs have been implicated in regulating the development and metastasis of human cancers. MiR221 is reported to be an oncogene in multiple cancers, including bladder cancer, breast cancer and thyroid cancer tumor cells [14–16]. The high expression of miR221 was correlated with the proliferation, invasion, and malignancy of thyroid cancer tumor cells [15]. The miR221/ZEB1 activity is efficiently targeted upon MEK1 inhibitor (TAK-733) treatment and when combined with irradiation treatment, significant reduction in migration of breast cancer cells was shown [16]. Likewise, in our study, miR221 expression was significant altered, and bioinformatic analysis revealed that *annexin a1* might be a target for miR221 regulation. As a matter of fact, *annexin a1* was individually validated as a miR-221 target in SNU-398, HepG2, and HEK293 cell lines [17].

Therefore, in the current study, as a continuation of our previous study, MeT-5A cells were continuously subjected to MWCNT exposure for up to 1 year at a sub-toxic dose. Finally, the MeT-5A cells was neoplastic-like transformed in vitro (named as MCN-MeT-5A), marked by increased proliferation and migration capacity, as well as anchorage-independent growth. Furthermore, the molecules responsible for the enhanced cell migration, especially ANNEXIN A1 and miR221, were investigated, in an effort to uncover the carcinogenic potential and possible mechanisms of MWCNT.

## Materials and methods

### MWCNT preparation

MWCNT (Aldrich 659,258) were purchased from Sigma-Aldrich (St Louis, MI) and stored at room temperature in darkness. Their main characteristics have been previously described and summarized [10, 18]. Briefly, the dimensions (diameter X length) were (110 nm–170 nm) X (5 μm − 9 μm), the purity was more than 90% with metal contaminants, mostly iron (less than 0.1%), and the specific surface area was 130 m^2^/g. The sterile raw material were weighed and suspended in sterile phosphate-buffered solution (PBS) to make a 5 mg/mL stock solution. In consideration of the bacterial endotoxin or lipopolysaccharides contamination [19], the stock solution underwent autoclaved sterilization and sonication before use, and diluted in cell culture medium to make a final concentration of 50 μg/mL (equals to 10 μg/cm^2^). The freshly sonicated stock solutions were prepared and visualized under field emission scanning electron microscopy (SEM) as previously described. The MWCNT’s microstructure in the solution was rigid and rod-like fiber with some occasional agglomerates, and the estimated diameters of MWCNTs ranged from 120 nm to 280 nm, and the estimated length ranged from 2 μm to 10 μm [18].

### Cell culture and transformation

Immortalized human pleural mesothelial MeT-5A cells (CRL-9444, American tissue culture collection ATCC) were routinely subcultured in M199 culture medium containing 10% fetal bovine serum at 37 °C and 5% CO_2_. For cell transformation, 1.5 X 10^5^–2 X 10^5^ per well MeT-5A cells were seeded into 6-well plates in triplicate and cultured overnight. The following day cells were continuously exposed to 10 μg/cm^2^ MWCNT for 48 h. The dose and exposure time of MWCNT were chosen based on the lactate dehydrogenase release (LDH) assay result in our previous study [10]. Then the old medium was removed, the adherent cells were gently rinsed in PBS for three times, and fresh M199 medium without MWCNT was administered. Basically, cells were passaged every 5–6 days by trypsinization, and the cycle lasted for 1 year. The attached cells were rinsed prior to culture medium changes and cell passages, in order to reduce potential accumulation of MWCNT over the exposure time course. PBS-exposed cells were used as controls and underwent all other the same treatments except for MWCNT-exposure. The cell morphology images were recorded by an inverted phase contrast microscope (Leica, Wentzler, Germany) if needed, and the cell number was counted by hemocytometer during the long-term treatments. The successfully neoplastic-like transformed cells after 1 year exposure were named as MCN-MeT-5A.

### Cell proliferation measurement

Cells (1.0 X 10^5^ cells) were seeded in wells of a 12-well plate (Corning) and grown in 3 mL media. After the indicated treatment, cells were trypsinized and pelleted by centrifugation at 300 g for 10 min. The pellet was then resuspended in culture medium. 50 μL of the cell suspension was diluted with an equal volume of Trypan blue solution (0.4%) (Sigma) and incubated for 3 min at room temperature. Cells were then loaded into a hemocytometer and analyzed for dye intake. The number of blue (dead) and white (live) cells were counted by an inverted microscope (Olympus, CKX31, Japan). The viability of both types of cells (original and MWCNT-treated) was calculated as: number of viable cells (white)/total number of cells (blue+white) X 100%.

### Soft-agar assays

The soft agar colony formation assay was performed according to the instructions with slight modification [20]. Briefly, 3 mL 0.7% bottom layer of agar was plated in 6-well plates and allowed to solidify at room temperature. Then passage-matched MeT-5A and transformed MCN-MeT-5A cells (1 X 10^4^ cells) seeded on 0.35% upper layer of agar were placed on it. The cell/agar mixture was placed into the 37 °C humidified cell culture incubator for about 2 weeks − 3 weeks. 100 μL of M199 medium was added over the upper layer of agar twice weekly to prevent desiccation. Cell colonies were observed and counted during 2 weeks − 3 weeks’ incubation.

### MicroRNA profiling

Illumina’s miRNA expression platform v2 (Cat. no. MI-102-1024; Illumina, San Diego, CA) was used to screen the differential expression of miRNAs in MeT-5A treated with MWCNT. This platform included 1146 unique human miRNAs based on the miRNA repository miRBase 12.0 (http://www.mirbase.org/). About 100 ng total RNA was polyadenylated and reverse transcribed into cDNA, amplified, and labeled by PCR. The PCR products were then hybridized to Sentrix Array Matrix. The array intensity data were collected and imported into BeadStudio v3.2 (Illumina), a software package that permits visualization and normalization of the data. After correction and normalization, the array intensity data with detection *P* values were analyzed according to the manufacturer’s recommendations. RNA isolation and quantitative real-time polymerase chain reaction (q-PCR).

Total RNA was extracted from cells using RNAiso Plus (Takara, Kusatsu, Shiga, Japan) according to the manufacturer’s instruction. miRNA was isolated with a mini miRNeasy kit (Qiagen, Hilden, Germany). Reverse transcription of 500 ng total RNA to cDNAs was performed with a miScript II RT Kit (Qiagen), and the cDNA was used as a template to amplify miR221 and *annexin a1* mRNA. qPCR was performed with a miScript SYBR Green PCR Kit (Qiagen) on an ABI 7500 fast Instrument (Applied Biosystems, Foster City, CA). Reverse transcription of 500 ng total RNA isolated from MCN-MeT-5A cells transfected by miR221 mimics was performed with a PrimeScriptTM RT reagent Kit (Takara) to cDNAs, and cDNAs were used as templates. The primers for human *annexin a1* mRNA were 5′-AGG GCC TTG GAA CTG ATG AA-3′ and 5′- CGG TCA CCC TTA GCA AGA GA-3′. mRNA expression was normalized against *GAPDH*. The primers for human *GAPDH* were 5′- TCA AGA AGG TGG TGA AGC AGG − 3′ and 5′- TCA AAG GTG GAG GAG TGG GT − 3′. The miR221 expression level was normalized to U6 RNA. Normalized gene expression levels were quantified to the respective controls. The relative expression was calculated using the comparative formula 2^-ΔΔCt^. Triplicate was set up for each sample, and each experiment was repeated at least twice.

### Transfection of cells with miR221 mimics

In the 6-well cell culture plate, 2 X 10^5^ MCN-MeT-5A cells were plated in 2 mL M199 medium with 10% serum. The next day, cells were transfected with 67 nM of miR221 mimics or non-targeting negative control mimics (NC) by using lipofectamine 3000 (Invitrogen, Carlsbad, CA) in a total 1.5 mL of M199 medium following the manufacturer’s protocol. Transfected cells were harvested at indicated time points to quantitatively analyze miR221 and *annexin a1* expression.

### Modulation of ANNEXIN A1 expression

To investigate the role of ANNEXIN A1 on cell migration, downregulation of ANNEXIN A1 was conducted by transfection with siRNA against *annexin a1* in MCN-MeT-5A cells. Briefly, MCN-MeT-5A cells were transfected with 10 nM siRNA to *annexin a1* (GenePharma) by using lipofectamine 3000 reagent (Invitrogen) in opti-MEM medium (Gibco), and incubated for 6 h at 37 °C. siRNA sequence to *annexin a1* is as below: 5′- GCC AUG AAA GGU GUU GGA ATT-3′. To exclude the possibility of off-target effects, cells were transfected with 10 nM non-target siRNA (GenePharma) as control.

To up-regulate the expression of ANNEXIN A1, cells were transfected with PYR-adshuttle-4 plasmid containing *annexin a1* CDS using the lipofectamine 3000 reagent (Invitrogen). The expression of ANNEXIN A1 was confirmed by western blot.

### Protein extraction and western blot analysis

Cells were lysed with RIPA lysis buffer (Beyotime) supplemented with Phenylmethanesulfonyl fluoride (PMSF) (Beyotime) and phosphatase inhibitor complex (Sangon Biotech, China) on ice for 40 min. The supernatant was collected after centrifugation at 10,000 g and 4 °C for 15 min. The protein concentration in the supernatant was measured by the BCA protein assay (Bio-Rad).

Equal amounts of protein were loaded and separated by 10% dodecyl sulfate sodium salt-Polyacrylamide gel electrophoresis (SDS-PAGE), and then transferred to polyvinylidene fluoride (PVDF) membranes in transfer buffer (25 mM Tris, 200 mM Glycine, 20% Methanol v/v). The membranes were blocked with 5% BSA in TBST (Tris 20 mM, NaCl 137 mM, Tween-20 0.1%, pH 7.6) for 1 h at room temperature. After washing with TBST, the membranes were incubated in primary antibody at 4 °C overnight followed by three washes with TBST and incubation with the secondary antibody for 1 h at room temperature. Antibodies against ANNEXIN A1 (BD, diluted 1:1000) and secondary antibodies HRP IgG (Multisciences, diluted 1:5000) were used. GAPDH (Santa Cruz, diluted 1:3000) was employed as an internal control. The protein bands were scanned using a FluorChem FC2 imaging system (Alpha, San Antonio, USA).

### Wound healing assay

To evaluate cell migration, a wound-healing assay was performed. 2 X 10^5^ cells per well were plated in the wells of a 6-well culture plates overnight, then cells were exposed to MWCNT treatments. When cells grew to confluence, the cell monolayer was scratched to form a 100-μm “wound” using sterile pipette tips and washed gently once with PBS. Cells were then incubated with regular medium for another 24 h and 48 h. The wound was photographed at 0, 24 h and 48 h using a DMI4000B microscope (Leica, Wentzler, Germany). The cell migration distance was measured by Image J software (National Institute of Mental Health, Bethesda, USA) at each time point. The ratio of the reduction of width at each time point to the initial width of scraped area (0 h) was expressed as percentage of migration (% migration rate) at each time point.

### Statistical analysis

Each experiment was conducted at least three times. Statistical analysis was performed using one-way ANOVA and Student’s t-test. Numerical values are represented by mean ± SD. A statistical probability of *P* < 0.05 was considered significant.

## Results

### Neoplastic-like transformation of MeT-5A cells after long-term MWNCT exposure

**Fig. 1:**
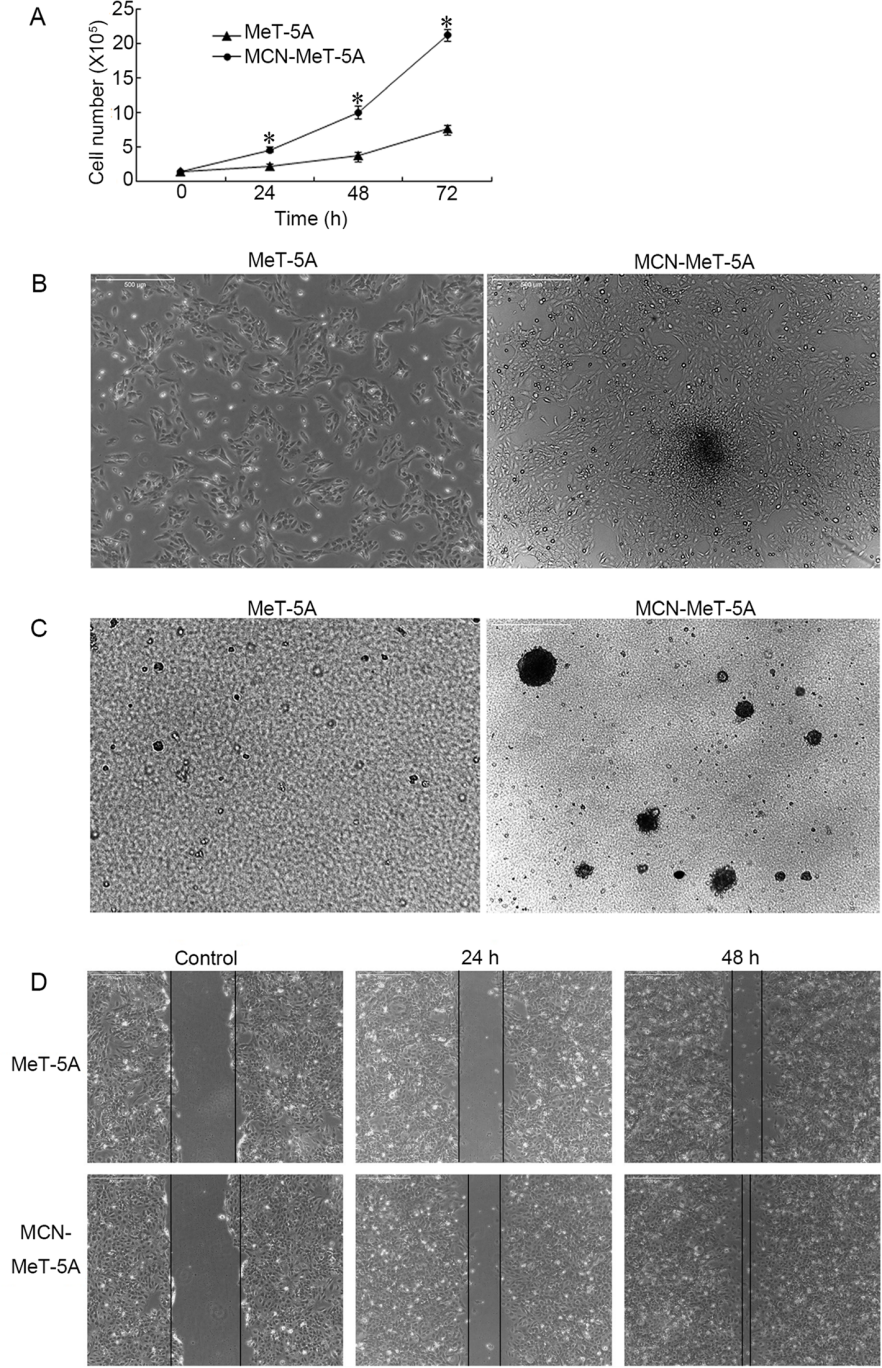
Characterization of neoplastic-like transformed MCN-MeT-5A cells. MeT-5A cells were chronic exposed to MWCNT at 10 μg/cm^2^ for one year as described in the Materials and Methods to generate the MCN-MeT-5A cells. **A** MCN-MeT-5A cells exhibited increased proliferation compared to the parental MeT-5A cells. Cell number was counted by hemocytometer. The number counting was repeated at least three times, and error bar stands for standard deviation (SD). *P < 0.05 versus control cells. **B** Cell mounds was formed in MCN-MeT-5A cells. The cell morphology images were recorded using a Leica microscope. Bar = 500 μm. **C** Soft agar colony formation assay showed multiple colonies formed by MCN-MeT-5A cells but not the passage-matched control MeT-5A cells. Bar = 500 μm. **D** MCN-MeT-5A cells migrated faster than passage-matched control MeT-5A cells in the wound healing assay at both 24 h and 48 h after scratching. Bar = 500 μm

MeT-5A cells were continuously exposed to MWNCT at 10 μg/cm^2^ for 1 year. At the end of one-year exposure, the resultant cells were named MCN-MeT-5A, and the proliferation capacity of these cells was compared to passage-matched MeT-5A cells. As shown in Fig. 1a, MCN-MeT-5A cells showed a dramatic increase in cell proliferation above controls, and the cell number was almost three times over control at 72 h, however, the viability of the original MeT-5A and MCN-MeT-5A remained almost the same (~ 90%, data not shown).

Morphologically, compared to the original MeT-5A cells, MCN-MeT-5A cells changed from the bigger, more spread-out shape to the smaller, tighter shape. More importantly, they lost the contact-inhibition, one of the most important indication of neoplastic-like transformation, and formed cell mounds (Fig. 1b). Furthermore, by using the colony-formation assay to evaluate anchorage-independent growth, another in vitro indicator commonly used for cell transformation, it was found that after incubation for 2 weeks, more than 10 colonies per well were formed in MCN-MeT-5A cells, while there was no colony formed in the passage-matched MeT-5A cells (Fig. 1c). In addition, cells picked up from the colony can be seeded into new plate and continuously cultured as well, indicating that they maintained the neoplastic property.

Finally, cell migration ability was also compared between MeT-5A and MCN-MeT-5A cells. As seen in Fig. 1d, MCN-MeT-5A cells clearly migrated much faster than control cells at both time points, indicating enhanced cell mobility for the transformed cells.

### The expression pattern of miR221 and ANNEXIN A1 during the long-term MWCNT exposure

**Fig. 2:**
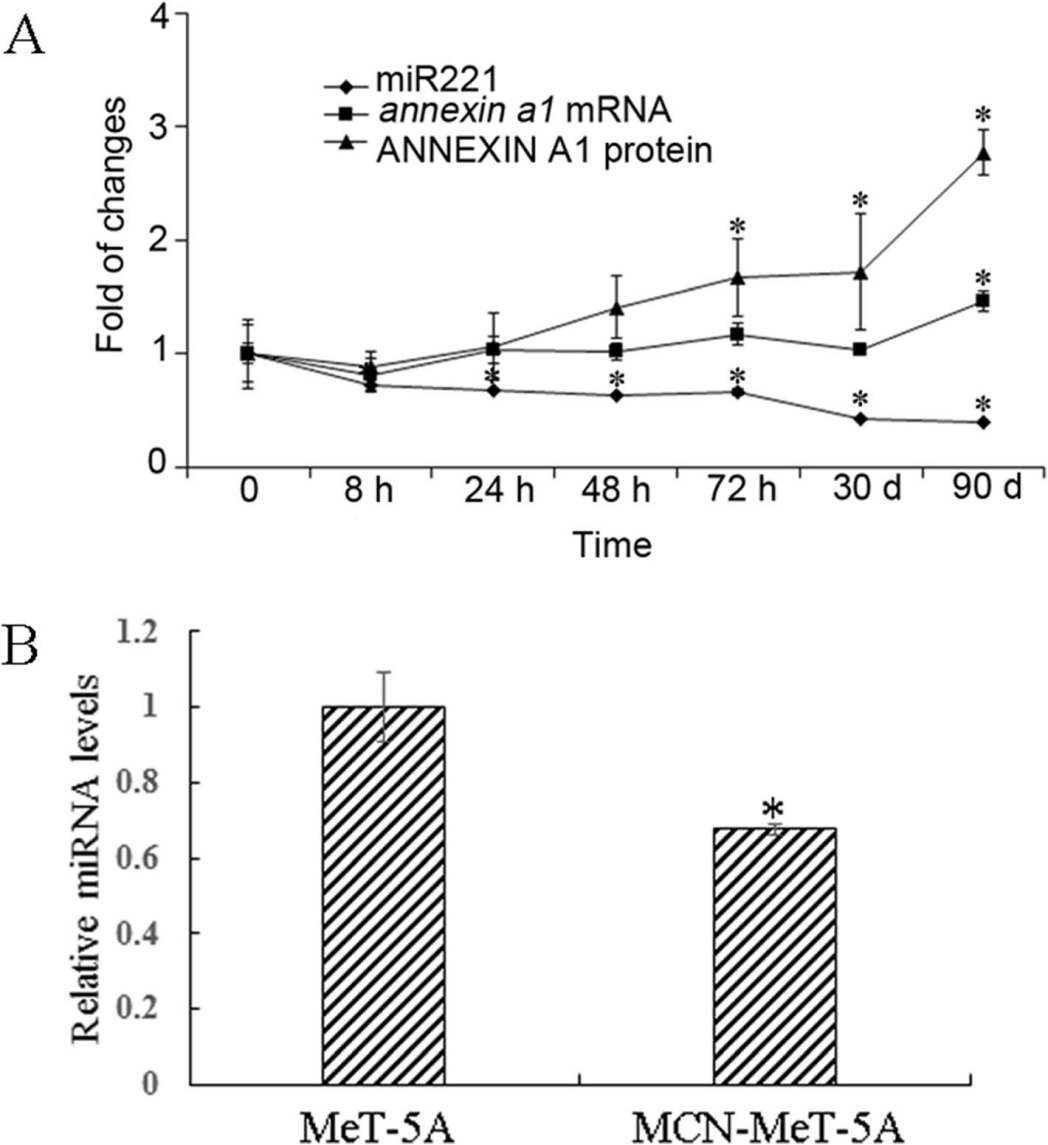
Expression of *annexin a1*, ANNEXIN A1 and miR221 in MeT-5Aafter MWNCT treatment. **A** MeT-5A cells were exposed to MWCNT at 10 μg/cm^2^ for up to 3 months, ANNEXIN A1 protein expression was detected by WB, while *annexin a1* mRNA and miR221 were examined by RT-PCR at the indicated time points. Each experiment was repeated at least three times, and error bar stands for standard deviation (SD). *P < 0.05 versus time 0. **B** Verification of miR221 expression by RT-PCR in MCN-MeT-5A cells after the miRNA profiling array. *P < 0.05 versus control cells

As mentioned earlier, ANNEXIN A1 might be involved in regulating cell migration, and miR221 might regulate *annexin a1* expression, therefore, the expression of these two molecules was examined in MWCNT-treated cells. Firstly, it was found that *annexin a1* expression showed an increasing trend over a three-month period at both mRNA and protein level, while the expression of miR221 exhibited a decreasing trend, showing a negative correlation between the two molecules (Fig. 2a).

A miRNA profiling microarray analysis was then conducted between MeT-5A and MCN-MeT-5A cells, to make it more clear the regulation of ANNEXIN A1 in our study. Some of the miRNAs identified with changed expression, including miR221 were shown in Table 1, and further verification of miR221 expression by RT-PCR showed the same trend (Fig. 2b).

**Table 1:** Some of miRNAs with expression changes after MWCNT treatment

Name	MeT-5A	MCN-MeT-5A	Fold change
hsa-miR-221	8.390829	5.8256145	0.694284
hsa-miR-28-5p	5.6068225	3.2131236	0.573074
hsa-miR-324-5p	6.282712	4.994509	0.794961
hsa-miR-10a	6.865937	2.631548	0.383276
hsa-miR-1281	2.115193	4.019866	1.900472

**Fig. 3:**
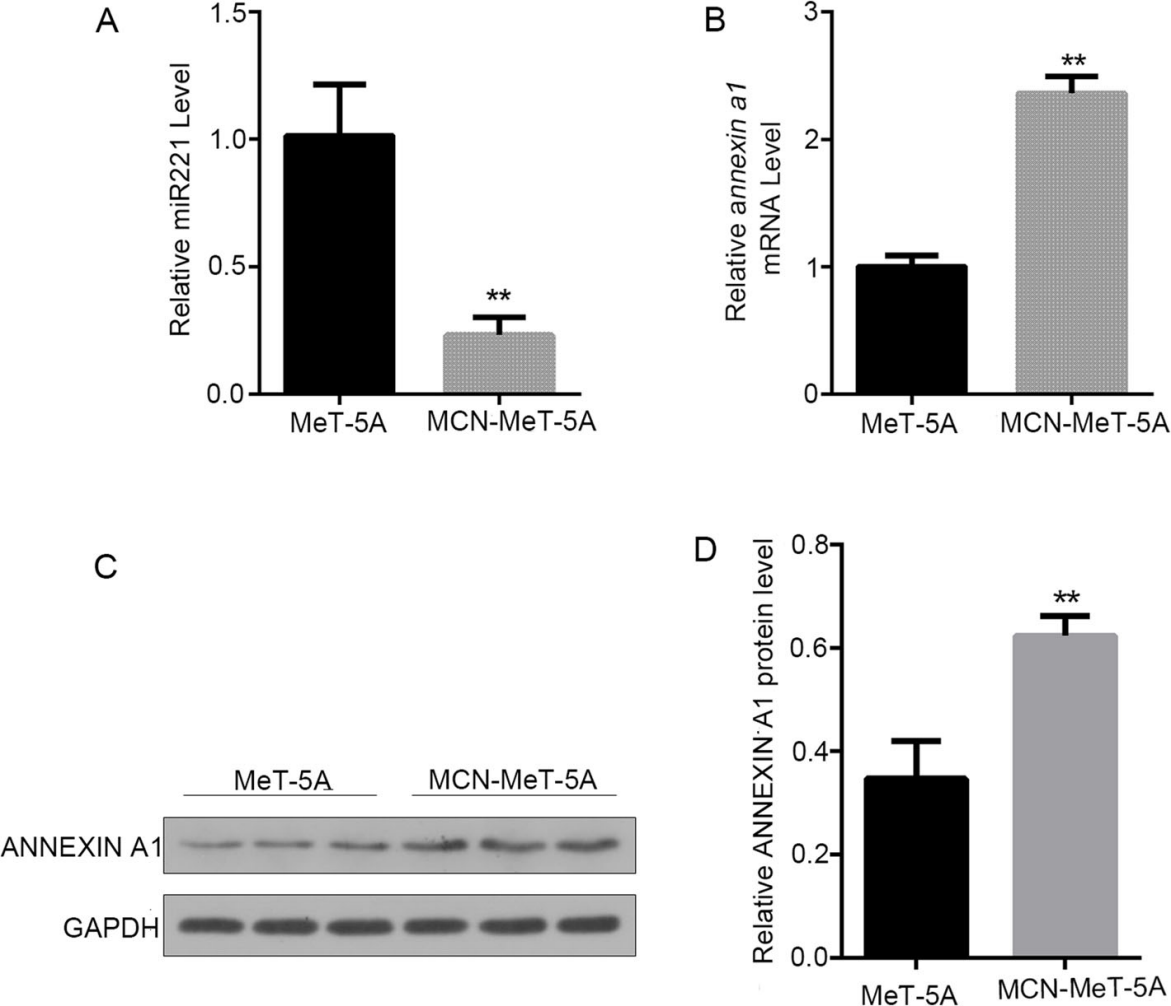
Expression of ANNEXIN A1 protein, *annexin a1* mRNA, and miR221 in MCN-MeT-5A Cells. ANNEXIN A1 protein expression was detected by WB, *annexin a1* mRNA and miR221 were examined by RT-PCR. Each experiment was repeated at least three times, and error bar stands for standard deviation (SD). ** *P* < 0.01 versus passage-matched control MeT-5A cells. **A** Relative mi221 level in MCN-MeT-5A and passage-matched MeT-5A cells. **B** Relative *annexin a1* mRNA level in MCN-MeT-5A and passage-matched MeT-5A cells. **C** ANNEXIN A1 protein level in MCN-MeT-5A cells and passage-matched MeT-5A cells. **D** Densitometry analysis of the results from (**C**)

The expression levels of ANNEXIN A1 and miR221 were also measured in MCN-MeT-5A cells. Compared with the passage-matched control MeT-5A cells, the ANNEXIN A1 protein and *annexin a1* mRNA levels were both significantly higher, while the expression of miR221 was much lower in MCN-MeT-5A cells (Fig. 3).

### Negatively regulation of miR221 to the expression of *annexin a1*

**Fig. 4:**
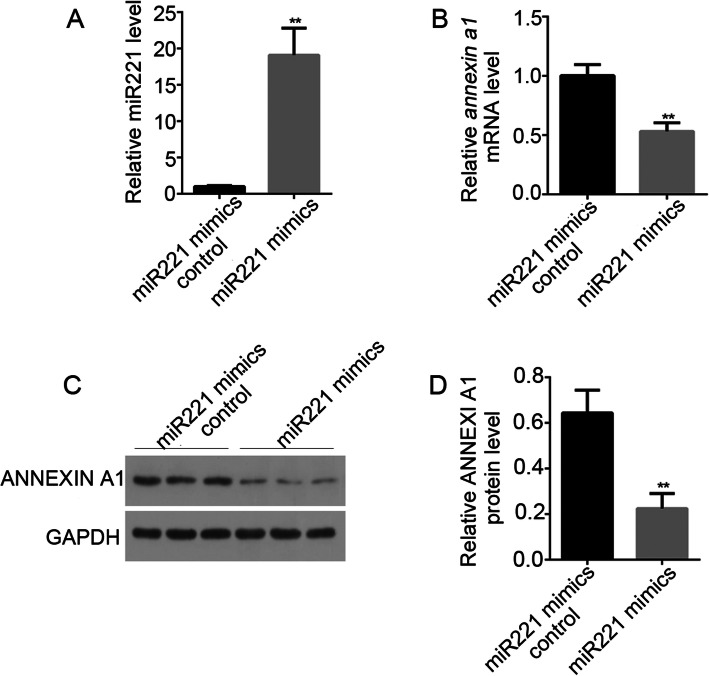
Effects of miR221 on *annexin a1* expression in MCN-MeT-5A Cells. Cells were transfected either with miR221 mimics or control sequence, and *annexin a1* expression was examined at both mRNA and protein level. Each experiment was repeated at least three times, and error bar stands for standard deviation (SD). ** *P* < 0.01 versus passage-matched control cells. **A** Relative miR221 level after transfection. **B** Relative *annexin a1* mRNA level after transfection. **C** WB examination of ANNEXIN A1 protein expression after transfection. **D** Densitometry analysis of the results from (**C**)

To further analyze the relationship between miR221 and *annexin a1*, we transiently transfected MCN-MeT-5A cells with a miR221 mimic. As expected, transient transfection with the specific miR221 mimic led to a robust increase of miR221 level. More importantly, this was accompanied by a drastic decrease in both the *annexin a1* mRNA and protein expression, as shown in Fig. 4. Thus, these results indicated a negative regulation of *annexin a1* by miR221.

### Effects of miR221 or ANNEXIN A1 modulation individually on cell migration of MCN-MeT-5A cells

**Fig. 5:**
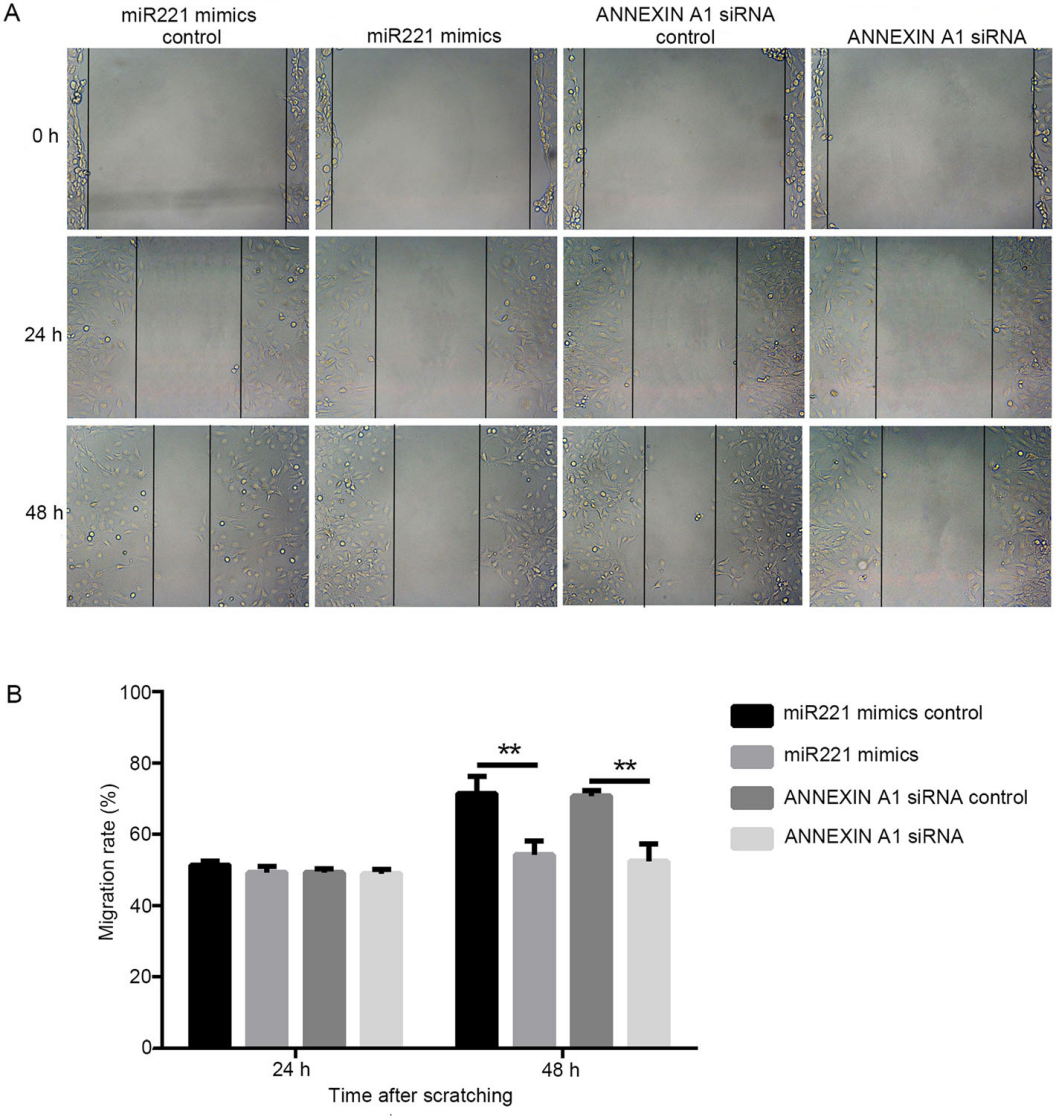
Effects of miR221 and the ANNEXIN A1 on cell migration of MCN-MeT-5A cells. **A** Cells were transfected with respective vectors as indicated, then were grown to confluence, scratched, and allowed to recover for 48 h. Shown are representative images of cell migration from three independent experiments (X100-fold, Scale bar = 500 μm). **B** Quantitative analysis of the results from (**A**). ** *P* < 0.01

To clarify the contribution of miR221 and ANNEXIN A1 to cell migration in the neoplastic-transformed cells, we modulated the expression of them individually and the cell migration ability was examined using wound-healing assay. MCN-MeT-5A cells were transfected with either a miR221 mimic or the control sequence as described above. As shown in Fig. 5, although there was no obvious difference observed at 24 h after scratching between the miR221-transfected cells and control cells, a significantly reduction of cell migration at 48 h was found for the miR221 mimic-transfected cells. Such results suggested that the ectopic expression of miR221 inhibited cell migration. Since *annexin a1* is a possible target of miR221 regulation, we then analyzed the effect of ANNEXIN A1 on cell migration by knocking down its expression using siRNA as described before [10]. As expected, similar to the effect of ectopic expression of miR221, down-regulation of ANNEXIN A1 also significantly suppressed cell migration (Fig. 5), further validating its function in this process.

**Fig. 6:**
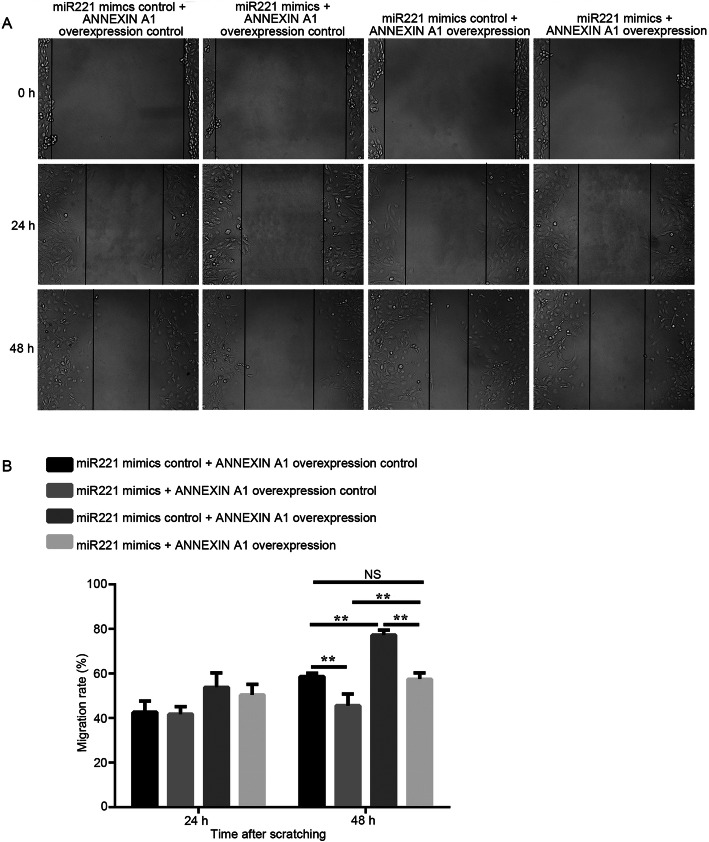
Effects of co-modulation of miR221 and ANNEXIN A1 on cell migration in MCN-MeT-5A cells. **A** Cells were transfected with the respective vectors as indicated, grown to confluence, scratched, and allowed to recover for 48 h. Shown are representative images of cell migration from three independent experiments (X100-fold, Scale bar = 500 μm). **B** Quantitative analysis of the results from (**A**). ** *P* < 0.01, NS stands for no significant changes

ANNEXIN A1 was overexpressed in MCN-MeT-5A cells by transfection with a PYR-adshuttle-4 plasmid containing *annexin a1* CDS, in an effort to further elucidate the interaction between *annexin a1* and miR221. Consistent with the above observation, the ectopic expression of miR221 inhibited cell migration, while the overexpression of ANNEXIN A1 promoted cell migration (Fig. 6). Interestingly, when cells were co-transfected with the miR221 mimic and the *annexin a1* overexpression plasmid, they counteracted each other, and thus had no effect on cell migration (Fig. 6).

## Discussion

The Stanton and Pott Hypothesis states that bio-persistent fibers in defined ranges of diameter and length cause cancer irrespective of their physicochemical nature, simply because they are fibers. This is known as the fiber paradigm and is an important consideration for safety assessment, particularly in the carcinogenicity of MWCNT. Actually, there is accumulating evidence that, like asbestos, inhaled nanomaterials of more than 5 μm long and with high surface-volume ratio (3:1), and particularly rod-like carbon nanotubes, may inflict pleural disease including mesothelioma [3, 21].

As a relatively common nanomaterial, the carcinogenesis potential of MWCNT has been investigated extensively over the years using different animal and cell models. Compared to the animal model, cells are usually used for mechanistic study with relatively short period (from hours to days) of exposure. Still, as the neoplastic transformation of cells is an important indicator for carcinogenicity, longer exposure of cells is also needed. For example, low dose sub-chronic MWCNT exposure of BEAS-2B cells up to 4 weeks induced an attachment-independent growth correlated with chromosome damage and reduced inflammatory signaling [22]. Human bronchial epithelial cells exposed to MWCNT (NM-400 and NM62002) can form colonies after 3 months [23]. Exposure of MeT-5A cells to MWCNT and single-walled carbon nanotube (SWCNT) for 4 months induced increased growth rate, migration, and invasion [24]. Chronic exposure (12 weeks − 24 weeks) to SWCNT caused neoplastic-like transformation of human lung epithelial cells [25]. Also, different cells may have different response, as Chortarea et al. reported that subchronic MWCNT exposure (5 weeks) in asthmatic cells revealed stronger and more durable long-term effects marked with an evident pro-inflammatory and oxidative stress response compared to healthy cells [26]. Consistent with the above studies, we have generated neoplastic-like transformed MeT-5A cells after 1 year exposure to MWCNT, characterized by increased cell proliferation, loss of contact inhibition, colony-formation ability, anchorage-independent growth, and increased migration, providing another piece of evidence for the possible carcinogenicity of MWCNT.

One important feature of the transformed cells, MCN-MeT-5A cells, is the increased cell migration. In a previous work [10], as well as in the current study, we reported that ANNEXIN A1 was significantly increased by MWCNT and it was closely related to increased cell migration. ANNEXIN A1 is a member of the ANNEXIN superfamily of calcium and phospholipid-binding proteins, which are involved in cell proliferation and differentiation, apoptosis, and intracellular signaling [27]. ANNEXIN A1 is also implicated in various types of human cancer. Elevated expression of ANNEXIN A1 predicts poor prognosis in human hepatocellular carcinoma and enhances cell malignant phenotype [28]. Upregulated ANNEXIN A1 promotes cellular invasion in triple-negative breast cancer [29]. Moreover, the increased ANNEXIN A1 at the tissue level is also found in various histological types of lung cancer compared with benign lung disease, especially in cancer tissues with poor differentiation and advanced stage, and there was a consistent trend of ANNEXIN A1 level among lung cancer tissue, bronchoalveolar lavage fluid (BALF) and serum [30].

In an attempt to identify the underlying mechanisms responsible for the increased expression of ANNEXIN A1 protein, we focused on its regulatory relationship with the miRNAs. MiRNAs are small non-coding RNAs that regulate gene expression post-transcriptionally by binding to the 3′-untranslated region (3′-UTR) of target mRNAs and induce the degradation or translation inhibition of that particular mRNA [31]. The exact role of miRNAs in MWCNT toxicity is less studied or understood, though there have reports showing changed expression of certain miRNAs by MWCNT exposure. For example, in bronchoalveolar lavage cells and lung granulomas, MWCNT increased miRNA33 expression [32]. Four miRNAs were correlated with MWCNT-induced mitochondrial membrane potential (MMP) suppression and one of them, miR1275, was found to be negatively correlated with a large part of the MMP suppression-associated genes [33]. MiRNAs (miR122/206/130/210) in the blood were significantly up- or down-regulated in mice with pathological alteration in the lung after methylcholanthrene administration followed by MWCNT inhalation [34]. MiR1 was suppressed by MWCNT after 6 or 24 h of treatment regardless of the dosage in alveolar epithelial A549 cells, and exogenous administration of miR1 induced cell morphology changes including cell clustering, whereas inhibition of miR1 induced less cell to cell contact, cell rounding, and cellular projections [35].

We also performed a miRNA profiling in MWCNT-exposed MeT-5A cells, in order to gain a global view of the miRNA response to MWCNT. Among the miRNAs identified with significant expression changes, miR221 was chosen as gene target prediction analysis suggested that *annexin a1* might be a direct target of miR221. Our results revealed that miR221 expression was indeed negatively correlated with *annexin a1* mRNA and ANNEXIN A1 protein level in MeT-5A cells after MWCNT treatments (Figs. 2 and 3). Moreover, ectopic expression of miR221 significantly down-regulated endogenous *annexin a1* mRNA and protein levels (Fig. 4). In our study, the functions of these two molecules in cell migration were evaluated, and the results clearly demonstrated a relationship between miR221 and *annexin a1* (Figs. 5 and 6).

The role of miR221 in cell migration has also been reported by others under different conditions. Downregulation of miR221 inhibits cell migration and invasion through targeting methyl-CpG binding domain protein 2 in human oral squamous cell carcinoma cells [36]. Additionally, miR221 is essential for the platelet-derived growth factor (PDGF)-mediated migration and growth of pancreatic cancer cells [37]. miR221 increases osteosarcoma cell proliferation, invasion and migration partly through the downregulation of PTEN [38]. In combination with these findings, our results point to the importance of miR221 contributing to the potential carcinogenicity of MWCNT.

Still, our study suffers some drawbacks. One key issue is that we did not thoroughly evaluate the possible effects of long-term culture on cells. We had conducted extra experiments to show whether one-year culture had any significant effects on MeT-5A cells: 1) observation of cell morphology; 2) trypan blue assay to evaluate cell proliferation; and 3) scratching assay to examine changes in cell migration. The results showed no significant changes in cell morphology, proliferation rate, and migration ability between the original MeT-5A cells and cells after 1 year passage, indicating that the long-term culture may not significantly affect the cells (Supplement Fig. 1 and 2). Nonetheless, whether any genetic drift in the cells occurred is not known. Furthermore, the mesothelial characteristics changes such as keratin expression, growth factor secretion, and even the ANNEXIN A1 and miR221 expression levels before and after long time sub-culturing are not compared. All these are important issues worth of future study and validation of our results.

## Conclusions

Long-term exposure of MWCNT in MeT-5A cells revealed a carcinogenic potential represented by cell transformation, with loss of contact-inhibition, an anchorage-independent growth, increased growth rate and cell migration. During this process, miR221-*annexin a1* axis plays vital role in regulating cell migration: the downregulation of miR221 resulted in increased expression of *annexin a1*, which in turn enhanced cell migration.

## Supplementary Information

**Additional file 1.**

**Additional file 2.**

## Data Availability

The datasets used and analyzed during the current study are available from the corresponding author on reasonable request.

## Abbreviations

MWCNT: Multi-walled carbon nanotube
miR221: microRNA 221
IARC: International agency for research on cancer
EMT: Epithelial-mesenchymal transition
DNA: Deoxyribonucleic acid
PTC: Papillary thyroid carcinoma
SCLC: Small cell lung cancer
miRNAs: microRNAs
PBS: Phosphate-buffered solution
SEM: Scanning electron microscopy
ATCC: American tissue culture collection
LDH: Lactate dehydrogenase release
qPCR: quantitative real-time polymerase chain reaction
NC: Negative control mimics
PMSF: Phenylmethanesulfonyl fluoride
SDS-PAGE: Dodecyl sulfate sodium salt-Polyacrylamide gel electrophoresis
PVDF: Polyvinylidene fluoride
SWCNT: Single-walled carbon nanotube
BALF: Bronchoalveolar lavage fluid
MMP: Mitochondrial membrane potential
PDGF: Platelet-derived growth factor
