# Supplementary figures and images for: miR221 regulates cell migration by targeting annexin a1 expression in human mesothelial MeT-5A cells neoplastic-like transformed by multi-walled carbon nanotube
Source: Genes Environ. 2021 Aug 2;43:34. doi: 10.1186/s41021-021-00209-y (PMC8327461; doi:10.1186/s41021-021-00209-y)

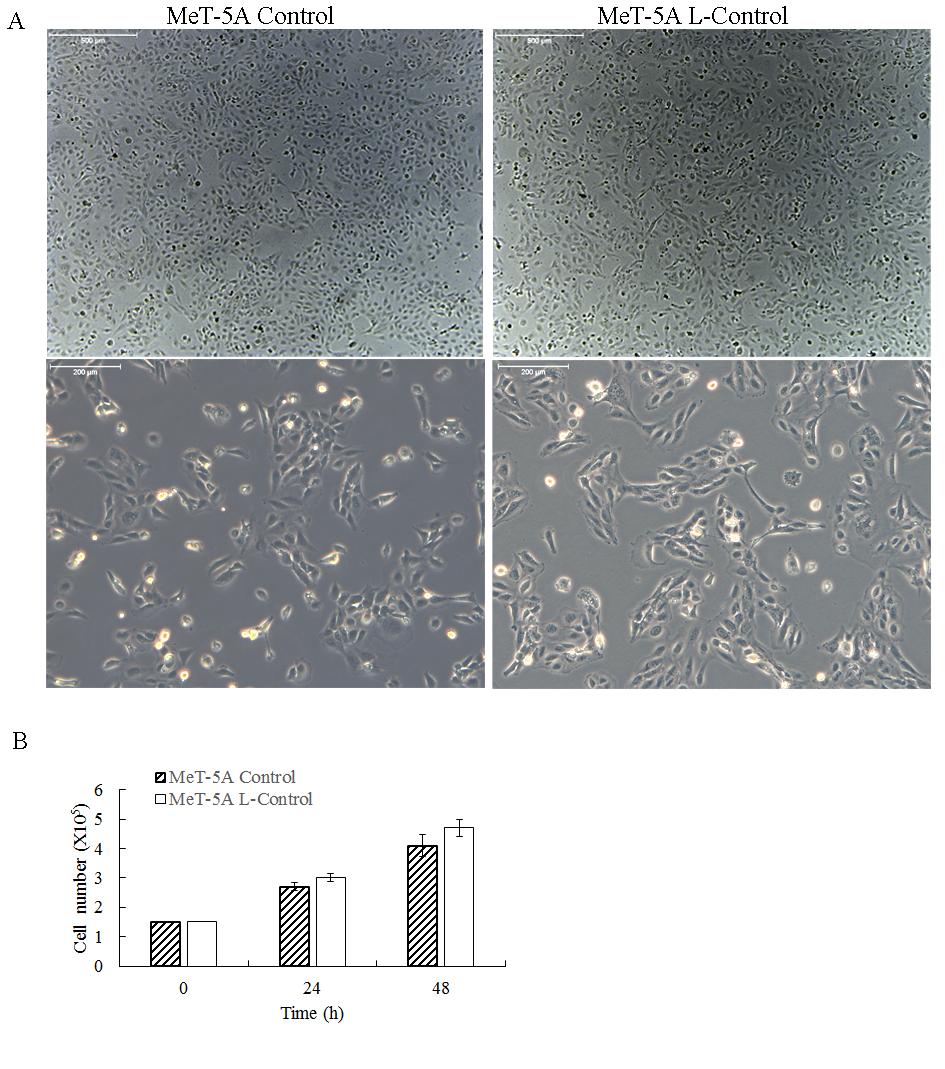

Supplement: Supplementary file 1 — Additional file 1. [file 41021_2021_209_MOESM1_ESM.tif]

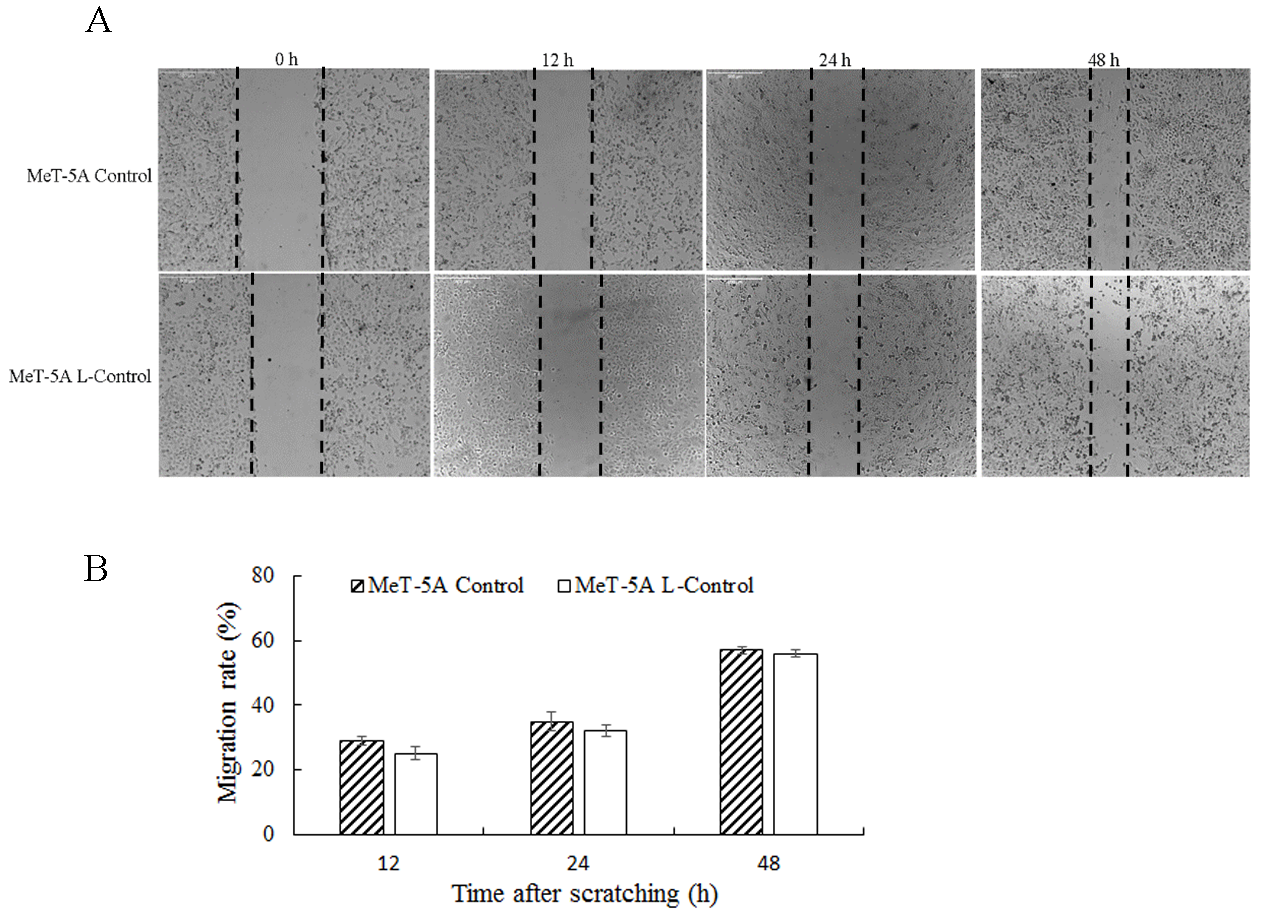

Supplement: Supplementary file 2 — Additional file 2. [file 41021_2021_209_MOESM2_ESM.tif]
